# Supplementary material for: Hyperuricemia and associated factors among adult cardiovascular disease patients at Salale University Comprehensive Specialized Hospital, Fitche, Central Ethiopia
Source: PLoS One. 2025 Jun 24;20(6):e0325775. doi: 10.1371/journal.pone.0325775 (PMC12186894; doi:10.1371/journal.pone.0325775)
Supplement: S1 Table — (DOCX) [file pone.0325775.s001.docx]

**Model fitness for SPSS Outputs**

| **Coefficients^a^** | | | | | | | | | | |
| --- | --- | --- | --- | --- | --- | --- | --- | --- | --- | --- |
| Model | | Unstandardized Coefficients | | Standardized Coefficients | t | Sig. | 95.0% Confidence Interval for B | | Collinearity Statistics | |
|  |  | B | Std. Error | Beta |  |  | Lower Bound | Upper Bound | Tolerance | VIF |
| 1 | (Constant) | -.473 | .494 |  | -.958 | .339 | -1.445 | .499 |  |  |
|  | Sex | .019 | .052 | .019 | .364 | .716 | -.083 | .120 | .826 | 1.211 |
|  | Residence | -.015 | .057 | -.015 | -.259 | .796 | -.126 | .097 | .674 | 1.485 |
|  | Education | .050 | .021 | .130 | 2.372 | .018 | .008 | .091 | .729 | 1.371 |
|  | Occupation | -.006 | .023 | -.014 | -.247 | .805 | -.051 | .039 | .644 | 1.553 |
|  | physical activity | .112 | .033 | .177 | 3.388 | .001 | .047 | .177 | .804 | 1.244 |
|  | Alcohol consumption | .063 | .032 | .111 | 2.012 | .045 | .001 | .125 | .724 | 1.381 |
|  | Cigarette | .236 | .115 | .101 | 2.043 | .042 | .009 | .463 | .895 | 1.117 |
|  | BMI | .039 | .039 | .060 | .986 | .325 | -.038 | .115 | .587 | 1.703 |
|  | centeral obesity | .136 | .066 | .126 | 2.069 | .040 | .007 | .265 | .591 | 1.692 |
|  | DM | .063 | .079 | .039 | .801 | .424 | -.092 | .219 | .919 | 1.088 |
|  | CKD | .201 | .059 | .185 | 3.437 | .001 | .086 | .317 | .758 | 1.320 |
|  | dyslipidemia | .121 | .061 | .104 | 1.965 | .050 | .000 | .242 | .787 | 1.271 |
|  | admission status | .164 | .054 | .167 | 3.065 | .002 | .059 | .270 | .746 | 1.341 |
|  | Duration | .005 | .028 | .009 | .177 | .860 | -.050 | .060 | .845 | 1.183 |
|  | chewing khat | -.097 | .186 | -.025 | -.522 | .602 | -.464 | .269 | .933 | 1.072 |
|  | age category | .067 | .050 | .068 | 1.347 | .179 | -.031 | .166 | .860 | 1.163 |
| a. Dependent Variable: hyperuricemia | | | | | | | | | | |


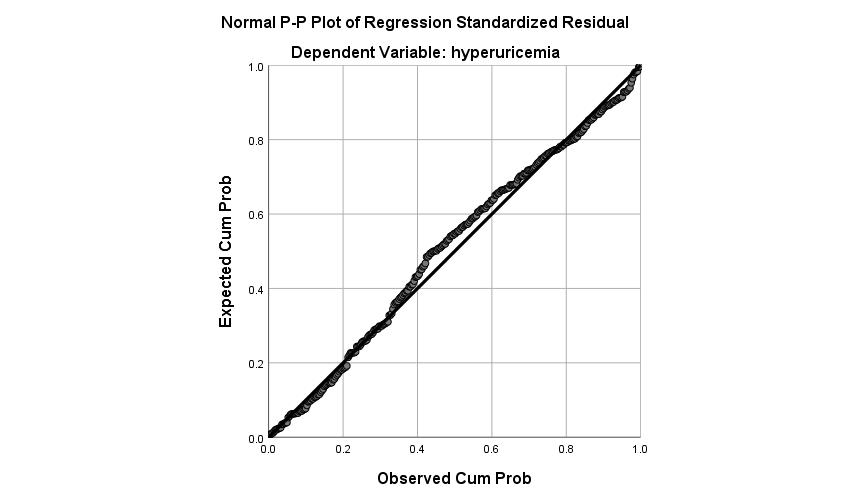


| **Omnibus Tests of Model Coefficients** | | | | |
| --- | --- | --- | --- | --- |
|  | | Chi-square | df | Sig. |
| Step 1 | Step | 165.221 | 38 | .000 |
|  | Block | 165.221 | 38 | .000 |
|  | Model | 165.221 | 38 | .000 |

| **Model Summary** | | | |
| --- | --- | --- | --- |
| Step | -2 Log likelihood | Cox & Snell R Square | Nagelkerke R Square |
| 1 | 238.774^a^ | .426 | .573 |
| a. Estimation terminated at iteration number 20 because maximum iterations has been reached. Final solution cannot be found. | | | |

| **Hosmer and Lemeshow Test** | | | |
| --- | --- | --- | --- |
| Step | Chi-square | df | Sig. |
| 1 | 11.096 | 8 | .196 |

| **Goodness-of-Fit** | | | |
| --- | --- | --- | --- |
|  | Chi-Square | df | Sig. |
| Pearson | 280.378 | 253 | .114 |
| Deviance | 250.339 | 253 | .535 |
