# Supplementary material for: Hyperuricemia and associated factors among adult cardiovascular disease patients at Salale University Comprehensive Specialized Hospital, Fitche, Central Ethiopia
Source: PLoS One. 2025 Jun 24;20(6):e0325775. doi: 10.1371/journal.pone.0325775 (PMC12186894; doi:10.1371/journal.pone.0325775)
Supplement: S2 Table — (DOCX) [file pone.0325775.s002.docx]

**Multinomial logistic regression result by SPSS**

| **Parameter Estimates** | | | | | | | | | |
| --- | --- | --- | --- | --- | --- | --- | --- | --- | --- |
| hyperuricemia^a^ | | B | Std. Error | Wald | df | Sig. | Exp(B) | 95% Confidence Interval for Exp(B) | |
|  |  |  |  |  |  |  |  | Lower Bound | Upper Bound |
| yes for male>7mg/dl and female>6mg/dl | Intercept | -3.602 | .948 | 14.443 | 1 | .000 |  |  |  |
|  | [Residence=1] | -.020 | .357 | .003 | 1 | .955 | .980 | .487 | 1.972 |
|  | [Residence=2] | 0^b^ | . | . | 0 | . | . | . | . |
|  | [Education=1] | .553 | .661 | .701 | 1 | .402 | 1.739 | .476 | 6.352 |
|  | [Education=2] | -.709 | .792 | .803 | 1 | .370 | .492 | .104 | 2.323 |
|  | [Education=3] | -.916 | .842 | 1.181 | 1 | .277 | .400 | .077 | 2.087 |
|  | [Education=4] | -.876 | .848 | 1.068 | 1 | .301 | .416 | .079 | 2.194 |
|  | [Education=5] | 0^b^ | . | . | 0 | . | . | . | . |
|  | [physical activity=1] | 1.415 | .488 | 8.420 | 1 | .004 | 4.116 | 1.583 | 10.704 |
|  | [physical activity=2] | .649 | .487 | 1.774 | 1 | .183 | 1.914 | .736 | 4.975 |
|  | [physical activity=3] | 0^b^ | . | . | 0 | . | . | . | . |
|  | [Alcohol consumption =1] | .763 | .403 | 3.585 | 1 | .058 | 2.144 | .974 | 4.723 |
|  | [Alcohol consumption =2] | .399 | .425 | .884 | 1 | .347 | 1.491 | .648 | 3.427 |
|  | [Alcohol consumption =3] | 0^b^ | . | . | 0 | . | . | . | . |
|  | [Cigarette=1] | 1.252 | .726 | 2.969 | 1 | .085 | 3.497 | .842 | 14.521 |
|  | [Cigarette=2] | 0^b^ | . | . | 0 | . | . | . | . |
|  | [BMI=1] | -.008 | .964 | .000 | 1 | .993 | .992 | .150 | 6.564 |
|  | [BMI=2] | .511 | .545 | .878 | 1 | .349 | 1.666 | .573 | 4.849 |
|  | [BMI=3] | -.073 | .394 | .035 | 1 | .853 | .929 | .430 | 2.010 |
|  | [BMI=4] | 0^b^ | . | . | 0 | . | . | . | . |
|  | [central obesity=1] | .703 | .455 | 2.382 | 1 | .123 | 2.019 | .827 | 4.928 |
|  | [central obesity=2] | 0^b^ | . | . | 0 | . | . | . | . |
|  | [History of DM=1] | .985 | .535 | 3.389 | 1 | .066 | 2.678 | .938 | 7.645 |
|  | [History of DM=2] | 0^b^ | . | . | 0 | . | . | . | . |
|  | [History of CKD=1] | 1.138 | .347 | 10.760 | 1 | .001 | 3.121 | 1.581 | 6.160 |
|  | [History of CKD=2] | 0^b^ | . | . | 0 | . | . | . | . |
|  | [history of dyslipidemia=1] | 1.012 | .398 | 6.461 | 1 | .011 | 2.751 | 1.261 | 6.004 |
|  | [history of dyslipidemia=2] | 0^b^ | . | . | 0 | . | . | . | . |
|  | [DCVD=1] | .458 | .647 | .502 | 1 | .479 | 1.581 | .445 | 5.617 |
|  | [DCVD=2] | .661 | .552 | 1.433 | 1 | .231 | 1.937 | .656 | 5.715 |
|  | [DCVD=3] | .415 | .531 | .610 | 1 | .435 | 1.514 | .535 | 4.287 |
|  | [DCVD=4] | 0^b^ | . | . | 0 | . | . | . | . |
|  | [age category=1] | .506 | .332 | 2.318 | 1 | .128 | 1.659 | .865 | 3.182 |
|  | [age category=2] | 0^b^ | . | . | 0 | . | . | . | . |
| a. The reference category is: no for male<=7mg/dl and female <=6mg/dl. | | | | | | | | | |
| b. This parameter is set to zero because it is redundant. | | | | | | | | | |

DCVD=duration of cardiovascular disease

BMI=Body Mass Index

BMI 1=Obesity, BMI 2=Over-weight, BMI 3=Normal weight, BMI 4=Under-weight

CKD=Chronic Kidney Disease

DM=Diabetic Mellitus
