# Supplementary material for: Hyperuricemia and associated factors among adult cardiovascular disease patients at Salale University Comprehensive Specialized Hospital, Fitche, Central Ethiopia
Source: PLoS One. 2025 Jun 24;20(6):e0325775. doi: 10.1371/journal.pone.0325775 (PMC12186894; doi:10.1371/journal.pone.0325775)
Supplement: Attachment — Submitted filename: Responses to reviewers.docx [file pone.0325775.s004.docx]

**Responses to reviewers**

**Dear Editors:** Thank you for your insightful feedback on our manuscript. We appreciate your time and expertise, which have been instrumental in strengthening the quality of our work. We have carefully addressed each of your comments and believe the revised manuscript now reflects these improvements. Our detailed responses to your specific points are provided below, indicated in ***italics*** for clarity

**Journal Requirements:**

1. Please review your reference list to ensure that it is complete and correct. If you have cited papers that have been retracted, please include the rationale for doing so in the manuscript text, or remove these references and replace them with relevant current references. Any changes to the reference list should be mentioned in the rebuttal letter that accompanies your revised manuscript. If you need to cite a retracted article, indicate the article’s retracted status in the References list and also include a citation and full reference for the retraction notice.

***Authors’ response:*** *We sincerely appreciate your attention to the matter of retracted references in our manuscript. Prior to submission, we diligently examined our citations for any retractions. This process included checks using both the Zotero citation management software and the Retraction Watch database. To further ensure accuracy, we also utilized an AI-powered tool to verify the absence of any retracted articles within our reference list. Despite these comprehensive efforts, we have found no evidence of retracted papers among our cited sources.*

2. Please consider and address all the comments to Revision1 version, done by the reviewers.

***Authors’ response:*** *Authors' Response: We sincerely appreciate your critical evaluation of our work. We have carefully addressed and incorporated each comment raised by both reviewers. We are grateful for the reviewers' comments and suggestions and will always welcome them.*

3. While revising your submission, please upload your figure files to the Preflight Analysis and Conversion Engine (PACE) digital diagnostic tool, [https://pacev2.apexcovantage.com/](https://track.editorialmanager.com/CL0/https:%2F%2Fpacev2.apexcovantage.com%2F/1/010f0195ea631273-e98b7b3b-320d-42e1-96c9-12aa8525922c-000000/ow25ya3BOiydy2R7cPv2Jpr6yCthn-ubNvy7B9_16ws=204). PACE helps ensure that figures meet PLOS requirements.

***Author’s response:*** *Thank you very much for the comments. We developed the figure, based on PACE tool.*

**Review Comments to the Author**

**Dear reviewers:** We sincerely appreciate your insightful and constructive feedback on our manuscript. Your comments have been invaluable in helping us to strengthen the clarity and rigor of our work. We have carefully considered each of your suggestions and have implemented revisions to address them. Our detailed responses to your specific points are provided below, indicated in italics for clarity.

**Reviewer #1**

1. ***Reviewer comment on second revision***: Abstract: Result: The highest prevalence of hyperuricemia was found among cardiovascular disease patients with congestive cardiac failure (16.2% - this is wrong, the correct number should be 48.0% as from Fig 2 chart) and hypertensive heart disease (15.8%, correct 41.2%).

***Authors’ response:*** *We thank you for your valuable comment. We have carefully considered it and incorporated the suggested changes into the revised manuscript****.***

***Reviewer comment on first revision Modified****:*

1. How would a patient with cardiomyopathy and heart failure be classified - as heart failure or cardiomyopathy?

***Authors’ responses****: We thank the reviewer for raising the important point regarding the subtypes of CVDs.*

- *In our study we considered heart failure and cardiomayopathy as distinct entities. We observed that congestive heart failure (CHF) – used almost synonymously with 'heart failure and it’s the predominant cardiovascular disease presentation [*[*https://www.heart.org/en/health-topics/heart-failure/what-is-heart-failure/types-of-heart-failure*](https://www.heart.org/en/health-topics/heart-failure/what-is-heart-failure/types-of-heart-failure)*]*
- *Likewise, while aware that cardiomyopathy includes specific subtypes like dilated cardiomyopathy (DCM), we strategically utilized the broader "cardiomyopathy" classification. This decision, supported by the referenced literature, allows us to maintain a clear focus on the most relevant cardiovascular disease subtypes within the scope of our research and provides the most accurate representation of the conditions we investigated as supported by this evidence : [*[*https://www.heart.org/en/health-topics/cardiomyopathy/what-is-cardiomyopathy-in-adults*](https://www.heart.org/en/health-topics/cardiomyopathy/what-is-cardiomyopathy-in-adults)*]*

2. I note that there are no patients with ischemic heart disease/coronary artery disease in the study population. Is this condition rare in the study population?

***Authors’ responses:*** *We sincerely thank the reviewer for highlighting the absence of a distinct category for coronary artery disease (CAD) or ischemic heart disease (IHD) within our cardiovascular disease (CVD) classification. We acknowledge the importance of considering these conditions. During data collection, we specifically sought to identify patients with CAD/IHD; however, we did not come across any patients presenting with these diagnoses. In our study area, CVD cases are typically categorized based on the presenting complication rather than the underlying etiology. The majority of patients with underlying CAD/IHD ultimately develop congestive heart failure (CHF), which was included as a distinct CVD category in our study. Therefore, while we recognize the etiological distinction of CAD/IHD, its absence as a separate category is due to the categorization practices within local clinical settings. Future studies in this region will incorporate a more detailed assessment of underlying CVD etiologies to address this limitation.*

**Reviewer #2**

***Reviewer comment***: Some terminologies and operational definitions (e.g., hyperuricemia, dyslipidemia, CKD) need to be more explicitly cited and consistently described across sections.

***Authors’ response:*** *We thank you for your valuable comment. We have revised and addressed into the revised manuscript****.***

***Reviewer comment***: The discussion of prevalence comparisons could benefit from additional explanation of population or methodological differences that may explain discrepancies with international literature.
There is still a need to tighten the language and reduce some redundancy, particularly in the discussion and background sections.

***Author’s response:*** *We greatly appreciate your thorough and insightful comments and suggestions. We have addressed each of these points comprehensively in our detailed response.*

**Detailed Comments**:

**Abstract:**

1. Reviewer comment: Clearly define how hyperuricemia was measured (thresholds by sex).

***Author’s response:*** *Thank you for the comments. We have acknowledged and incorporated this point into the abstract section of new revision of manuscript.*

2. Reviewer comment: Consider briefly including the hospital’s setting/context in the background sentence for international readers.

***Author’s response:*** *Thank you for the helpful comments. We have revised the abstract to reflect this point.*

**Introduction**

1. *Reviewer comment:* The background is comprehensive, but the rationale should be more focused on why studying hyperuricemia in the Ethiopian CVD population is urgent.

***Author’s response:*** *Thank you for the helpful comments. We have revised the abstract to reflect this point.*

2. *Reviewer comment:* Remove some repetitive sentences on uric acid pathophysiology and clinical consequences.

***Author’s response:*** *We are grateful to the reviewer for their valuable feedback, which has led to significant improvements in the manuscript. In particular, we have carefully addressed the concern regarding repetitive descriptions of uric acid pathophysiology and clinical consequences in new manuscript.*

**Methods:**

1. *Reviewer comment:* Please cite a specific reference for the dyslipidemia criteria from NCEP ATP III.

***Author’s response:*** *Thank you for your insightful and constructive comments. We acknowledge and have addressed your feedback in the revised manuscript, specifically in the Methods section on page [8].*

2. *Reviewer comment:* Mention whether quality control procedures for biochemical assays followed international guidelines (e.g., CLSI or WHO).

***Author’s response:*** *We are grateful for the reviewer's critical and insightful feedback. In performing our biochemical analyses using the Roche COBAS c 311, we prioritized adherence to the manufacturer's validated procedures and standards for calibration, quality control, maintenance, and patient sample analysis. This approach ensures consistency and traceability, particularly as Roche designs the analyzer to align with prominent international guidelines, including ISO 15189, CLSI, and ISO 13485. Our laboratory's SOPs for maintenance, QC, and calibration are also directly derived from the manufacturer's manual and guidelines. We believe that maintaining this level of consistency with the manufacturer's validated protocols was essential for the reliability and reproducibility of our results.*

**Results:**

1. *Reviewer comment:* Results are clearly presented.

***Author’s response:*** *Thank you very much for your positive feedback and recognition regarding the clarity of our results presentation!*

2. *Reviewer comment:* Figures and tables are useful. Ensure that all percentages are consistently reported with the same number of decimal points.

***Author’s response:*** *Thank you for your feedback. We have acknowledged and incorporated your suggestions by revising the Abstract and Results sections of the new manuscript.*

**Discussion:**

*1. Reviewer comment:* Briefly discussing dietary or genetic predispositions in Ethiopia that may influence uric acid levels would improve this section.

***Author’s response:*** *Thank you for your insightful and constructive comments. We acknowledge and have addressed your feedback in the revised manuscript with track, specifically in the discussion section on page [18].*

*2. Reviewer comment:* Strengthen the section on the implications for public health or clinical interventions based on your findings.

***Author’s response:*** *Thank you for your feedback. We have acknowledged and incorporated your suggestions by revising the discussion sections of the new manuscript with track change on page [19.20].*

**Limitations:**

*1. Reviewer comment* The limitation regarding diet and purine intake is appreciated and well discussed.

***Author’s response:*** *Thank you very much for your positive feedback and recognition regarding the clarity of our results presentation!*

*2. Reviewer comment*; Add a note that self-reported lifestyle variables (e.g., physical activity, alcohol consumption) may be subject to recall bias.

***Author’s response:*** *Thank you for the comments. We have acknowledged and incorporated this point into the limitation section of new revision of manuscript.*

**Conclusion:**

*1. Reviewer comment*; Concise and consistent with the findings. Emphasize the importance of screening and early management strategies in resource-limited settings.

***Author’s response:*** *Thank you for your feedback. We have acknowledged and incorporated your suggestions by revising the conclusion sections of the new manuscript.*
